# Decrease in hyperosmotic stress–induced corneal epithelial cell apoptosis by L-carnitine

**Published:** 2013-09-19

**Authors:** Neeta Khandekar, Mark D.P. Willcox, Sharon Shih, Peter Simmons, Joseph Vehige, Qian Garrett

**Affiliations:** 1 Brien Holden Vision Institute, Sydney, Australia; 2 School of Optometry and Vision Science, University of NSW, Sydney, Australia; 3 Allergan Inc, Irvine, CA

## Abstract

**Purpose:**

To characterize the osmoprotective properties of L-carnitine on human corneal epithelial cell volume and apoptosis during hyperosmotic stress.

**Methods:**

Human corneal limbal epithelial (HCLE) cells were exposed to culture medium at 300 mOsm (isotonic) or 500 mOsm (hyperosmotic) with or without L-carnitine (10 mM). Induction of apoptosis was detected by quantifying the proteolytic activity of caspase-8, caspase-9, and caspase-3/7 using caspase activity assays, the expression of tumor necrosis factor (TNF)-α with enzyme-linked immunosorbent assay, and annexin V/propidium iodide staining of HCLE cells evaluated with confocal microscopy and flow cytometry. Cell volume changes in response to hyperosmotic stress were analyzed using flow cytometry.

**Results:**

After the HCLE cells were exposed to hyperosmotic medium (500 mOsm), the percentage of shrunken cells and damaged/dead cells (stained positively for annexin V and/or propidium iodide) was six- and three-fold, respectively, higher than that under isotonic conditions (300 mOsm). This was paralleled by an increase in TNF-α concentration in media and caspase-8, -9, and -3/7 activities (six-, four-, ten-, and twelve-fold, respectively; all showing p<0.001). Addition of L-carnitine during hyperosmotic stress partly restored cell volume and significantly reduced the concentration of TNF-α released (p=0.005) and caspase-9 activity (p=0.0125). Addition of L-carnitine reduced the percentage of hyperosmolarity-induced damaged/dead cells to levels observed under isotonic conditions.

**Conclusions:**

L-carnitine can regulate human corneal epithelial cell volume under hyperosmotic stress and ameliorate hyperosmotic stress–induced apoptosis.

## Introduction

Hyperosmolarity is a feature common to many cases of dry eye disease, although there is sometimes a lack of correlation in studies between other dry eye tests and osmolality measurements of the tear film. Hyperosmolarity can result from either a decrease in tear secretion or an increase in tear evaporation—the two pathways that produce ocular dryness [1,2]. Tear osmolarity measured from the lower meniscus of tears of patients with dry eye can reach values of up to 360 mOsm [3,4] compared with 300–310 mOsm in normal eyes [5-7]. However, the osmolarity measured from the lower meniscus might not fully reflect osmolarity over the ocular surface. Although measuring the osmolarity directly over the ocular surface has remained technically challenging, it has been proposed that the tear film osmolarity in these regions can increase to 450 to 600 mOsm [8-10].

Tear hyperosmolarity is the central mechanism in the pathogenesis of ocular surface damage and is associated with inflammation in dry eye disease [2,11]. An increase in tear osmolarity has also been found to correlate with the severity of dry eye disease across normal, mild/moderate, and severe categories [12]. Hyperosmolarity-associated ocular surface damage and inflammation have been widely demonstrated in studies using animal dry eye models [13-16] as well as in in vitro human corneal epithelial cell culture models [17,18]. Tear hyperosmolarity can damage the surface epithelium, which can trigger production of signaling molecules, including various interleukins, tumor necrosis factor, and matrix metalloproteinases [13,14,17,19], as well as decrease the number of conjunctival goblet cells, which results in a disturbance of mucin expression leading to tear instability and subsequent increases in the ocular surface osmolarity, thus perpetuating the inflammatory cycle [20,21]. Although a direct relationship between high tear osmolarity and ocular surface damage has not been firmly established in human subjects, Reinoso et al. demonstrated significantly increased apoptosis levels in the conjunctival epithelium of patients with evaporative dry eye disease compared with normal eyes [22].

Exposure of the ocular surface to a hyperosmotic environment causes an imbalance between the extracellular and intracellular compartments resulting in net efflux of water from the ocular surface epithelial cells leading to cell shrinkage [17,18,23]. Excessive alterations in cell volume impinge upon cell survival, interfering with the cell membrane, cytoskeletal integrity, and cytosolic proteins [24]. To counteract these harmful effects, the cells respond through an immediate intracellular influx and accumulation of components including inorganic ions through activation of ion transporters, exchangers, or channels, which helps to equilibrate osmolality and thus regulate cell volume [24]. The survival of hypertonicity-stressed corneal epithelial cells depends on Na^+^ K^+^ 2Cl^-^ cotransporter (NKCC) activity, which is controlled by p38 mitogen-activated protein kinase (MAPK) activation [25-27]. However, the accumulation of inorganic ions in cells can interfere with normal cellular processes and cause precipitation of cell macromolecules, denaturation and destabilization of internal proteins, alterations in membrane potentials, and changes in the rates of enzymatic reactions, leading to premature cell death [24,28-32]. Hyperosmolarity-induced apoptosis in cultured human ocular surface epithelial cells has been reported [33-35].

Cells can adapt to the hyperosmotic environment by taking up organic osmolytes (also known as osmoprotectants) [36], which, unlike inorganic ions, do not interfere with cell metabolism or destabilize proteins, thus protecting against cellular damage and helping cells survive and function [37]. L-carnitine is a naturally occurring amino acid best known for its role in the mitochondrial oxidation of long chain fatty acids [38]. Carnitine also participates in cell volume and fluid balancing in all tissues affected by the tonicity (iso-, hyper-, hypotonicity) of the extracellular environment [39]. In the kidney, osmolytes including carnitine are crucial in maintaining cell volume in an environment of drastically fluctuating tonicity resulting from fluctuations in increased production of urine and antidiruesis. The resulting increased extracellular osmolarity of medullary cells can be more than four-fold that of isotonicity [40-42]. In the brain, the role of carnitine in isotonicity is crucial since alteration of tonicity affects nerve excitability due to ion fluctuation, and brain cells cannot swell due to skull rigidity [39]. In the ocular system, Corrales and colleagues [17] reported that under hyperosmolar conditions, L-carnitine protects cultured human corneal epithelial cells through its inhibition of hyperosmolarity-induced activation of mitogen-activated protein (MAP) kinases although the nature of the potential interaction of L-carnitine with signaling pathways for MAP kinases has not been explored. L-carnitine also protects rabbit corneal epithelial cells from hyperosmotic stress in dry eye models [43]. L-carnitine can also limit progression and reduce the severity of mouse dry eye with demonstrated reduction in corneal staining and several apoptotic epithelial cells [44]. In mammals, carnitine, obtained by in situ biosynthesis and from the diet [45], is maintained at a steady-state concentration in many tissues such as skeletal and heart muscles, principally by the organic cation transporter (OCT) system, specifically by OCTN1 and OCTN2 [46-48].

Previous studies demonstrated the presence of OCTN1 and OCTN2 in human and rabbit corneal and conjunctival epithelial cells [49]. These transporters are upregulated under hyperosmotic stress [49]. The level of L-carnitine in tears is depleted during dry eye [50], and there are reports that administering L-carnitine to the ocular surface improves the common signs and symptoms of dry eye and reduces dry eye–associated conjunctival staining [51].

In this study, we used an in vitro human corneal-limbal epithelial (HCLE) culture model to determine the osmoprotective properties of L-carnitine through its ability to regulate hyperosmolarity-induced cell volume changes and apoptosis characterized by the proteolytic activity of caspase-8, caspase-9, and caspase-3/7 and the release of tumor necrosis factor (TNF)-α. We provide evidence demonstrating that L-carnitine can aid in maintaining cell volume under hyperosmotic stress and ameliorate certain aspects of hyperosmotic stress-induced apoptosis.

## Methods

### Cell culture

An immortalized HCLE cell line derived from primary cultures of HCLE cells (a kind gift from Dr. Ilene Gipson, Schepens Eye Research Institute, Boston, MA) was used in the study. Cells were maintained at 2×10^4^/cm^2^ in a keratinocyte serum-free medium (K-SFM; Invitrogen-Gibco, Grand Island, NY), supplemented with 25 μg/ml bovine pituitary extract, 0.2 ng/ml epidermal growth factor (EGF; Invitrogen, Mount Waverley, Australia), and 0.4 mM CaCl_2_, and grown at 37 °C in a 5% carbon dioxide atmosphere. To enhance nutrient composition, the cultures were switched at approximately 50% confluence to a 1:1 mixture of K-SFM and low calcium Dulbecco's Modified Eagle Medium (DMEM)/F12 medium (Invitrogen) to achieve confluence. For the influence of Ca^2+^ on the cellular response to hyperosmolar stress and the effect of L-carnitine, cells were transferred to a 1:1 mixture of K-SFM and 1.0 mM Ca^2+^ DMEM/F12 medium to achieve confluence.

### Exposure of human corneal-limbal epithelial cells to hyperosmotic conditions in the presence of L-carnitine

A hyperosmolar medium of 500 mOsm was achieved with the addition of NaCl. Osmolality was confirmed using a Vapro 5520 vapor pressure osmometer (Wescor, Logan, UT). HCLE cells with 70%–80% confluence were exposed to isotonic (300 mOsm) or hypertonic media (500 mOsm) in the presence or absence of 10 mM L-carnitine [17] or 10 mM D-carnitine (as a comparison), for 16 h at 37 °C. The osmolarity and exposure time were established previously, based on our preliminary evidence indicating that the osmolarity of 500 mOsm and an exposure time of 16 h were optimal for studying measurable cellular responses [52].

### Caspase activity assay

Luminescent assay kits (Promega, Madison, WI) were used to measure the proteolytic activity of caspase-8, caspase-9, and caspase-3/7. The assays were performed according to the manufacturer’s instructions. Briefly, 100 µl of caspase-8, caspase-9, or caspase-3/7 luminogenic substrate reagents were added individually to HCLE cells after 16 h exposure to isotonic or hyperosmotic medium in the presence or absence of 10 mM L-carnitine. The reagents and cells were incubated at room temperature for 1 h after which the luminescence was measured using a Tecan SpectraFluor Plus 137 microplate reader (Tecan, Männedorf, Switzerland).

### Measurement of tumor necrosis factor-α production

Production of TNF-α by HCLE cells in the culture supernatants was evaluated using enzyme-linked immunosorbent assay (R&D Systems, Minneapolis, MN) according to the manufacturer’s instructions. The culture supernatants were collected, centrifuged at 239 ×g for 5 min to remove the detached cells, and stored at −80 °C until processed. Absorbance was measured at 540 nm using a Multiskan spectrum spectrophotometer (Thermo Scientific, Vic, Australia)

### Confocal microscopic evaluation of human corneal-limbal epithelial cells

Apoptosis or necrosis in HCLE cells was monitored with the Alexa Fluor 488 annexin V/propidium iodide (PI) assay (Vybrant apoptosis assay kit; Invitrogen, Australia) using a confocal microscope (FluoView FV1000 confocal laser scanning microscopy; Olympus, Tokyo, Japan). HCLE cells were seeded at a concentration of 6.5×10^4^ cells/ml in 75 cm^2^ cell culture flasks, and 70%–80% confluent cells were subjected to 300 mOsm and 500 mOsm media with and without 10 mM L-carnitine for 16 h. Total cells (the detached cells were collected after the media were spun at 239 ×g for 5 min and the remaining adherent cells were collected with trypsinization) were then stained with Alexa Fluor 488 Annexin V (5 µl/100 µl), Hoechst 33342 (12.5 µg/ml), and PI (1 µg/ml) for 15 min at room temperature, and washed with annexin-binding buffer (Invitrogen, Australia) before the confocal microscopic evaluation. Images of five different fields of view for each sample (three samples per treatment) were captured, and cells were quantified using ImageJ (National Institutes of Health, Bethesda, MD).

### Flow cytometry analysis of human corneal-limbal epithelial cell responses

Cell apoptosis or necrosis was further evaluated using an Attune Acoustic focusing flow cytometer (Life Technologies Applied Biosystems, Vic, Australia). Vybrant cell apoptosis kit (Invitrogen, Australia) containing annexin V and PI was used to analyze the physiologic state of the cells in response to 16 h exposure to isotonic (300 mOsm) or hypertonic (500 mOsm) conditions with or without 10 mM L-carnitine or D-carnitine. Staining was performed according to the manufacturer’s instructions. Annexin V and PI staining was evaluated using the blue/violet laser at excitation/emission of 480/500 nm (20 mW) and the blue/red laser at excitation/emission of 490/635 nm, respectively. Based on the principle that the forward scatter of the laser light produced by the flow cytometer is proportional to the volume of the cell, while the side scatter represented cells with greater structural complexity and granularity and thus a shrunken cell [53], the cell volume changes in response to isotonic (300 mOsm) or hyperosmotic (500 mOsm) challenge with or without 10 mM L-carnitine (16 h) were evaluated using flow cytometry by gating populations such that the cells with greater forward scatter constituted the percentage of cells with intact cell volume while those showing greater side scatter comprised the shrunken cell population.

### Data analysis

All results are presented as mean±standard deviation (SD). Group means were compared using one-way analysis of variance (ANOVA) with Bonferroni correction. The Student *t* test was performed to compare data comprising two groups at the p<0.05 level of significance.

## Results

### Caspase activity in response to hyperosmotic stress in the presence of L-carnitine

**Figure 1:**
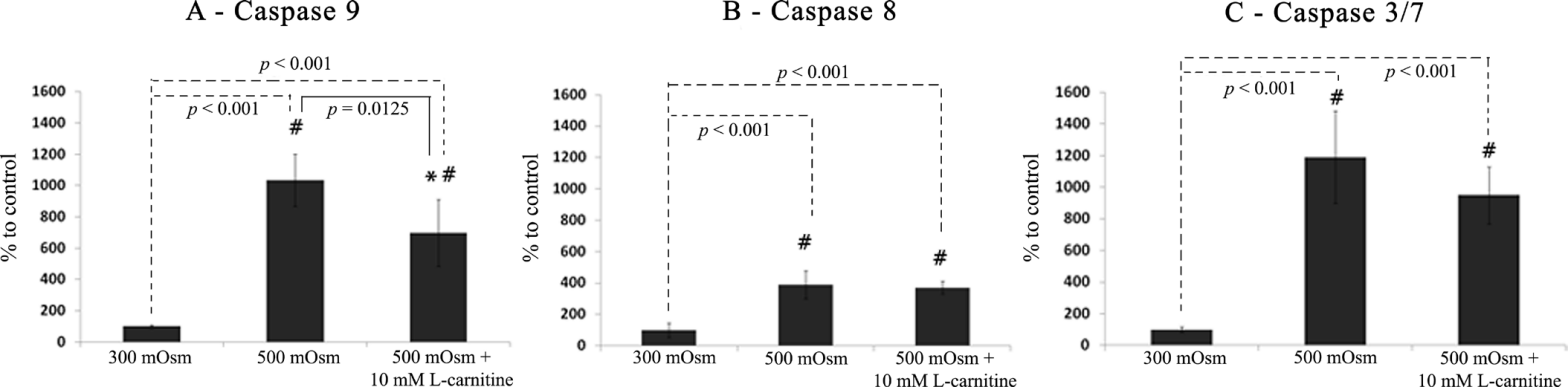
Activity of caspase-9 (**A**), caspase-8 (**B**), and caspase-3/7 (**C**) during isotonic (300 mOsm) or hyperosmotic (500 mOsm) stress in the presence or absence of L-carnitine (10 mM). Data are shown as the percentage mean±SD (n=6) over the isotonic medium group (300 mOsm). * shows the significant difference between cells treated with and without L-carnitine during exposure to 500 mOsm medium osmolarity. ^#^ shows the significant difference from 300 mOsm medium.

When exposed to the hyperosmotic medium (500 mOsm) for 16 h, the activity of caspase-8, -9, and -3/7 were increased significantly (each with p<0.001) with approximately four-, ten-, and twelve-fold of the value of isotonic cells, respectively (Figure 1). Adding L-carnitine (10 mM) during hyperosmotic stress suppressed elevation of caspase-9 activity significantly (Figure 1A; p=0.0125). However, no significant effect was observed for L-carnitine on caspase-8 (Figure 1B) and caspase-3/7 (Figure 1C).

### Tumor necrosis factor-α production in response to hyperosmotic stress in the presence of L-carnitine

**Figure 2:**
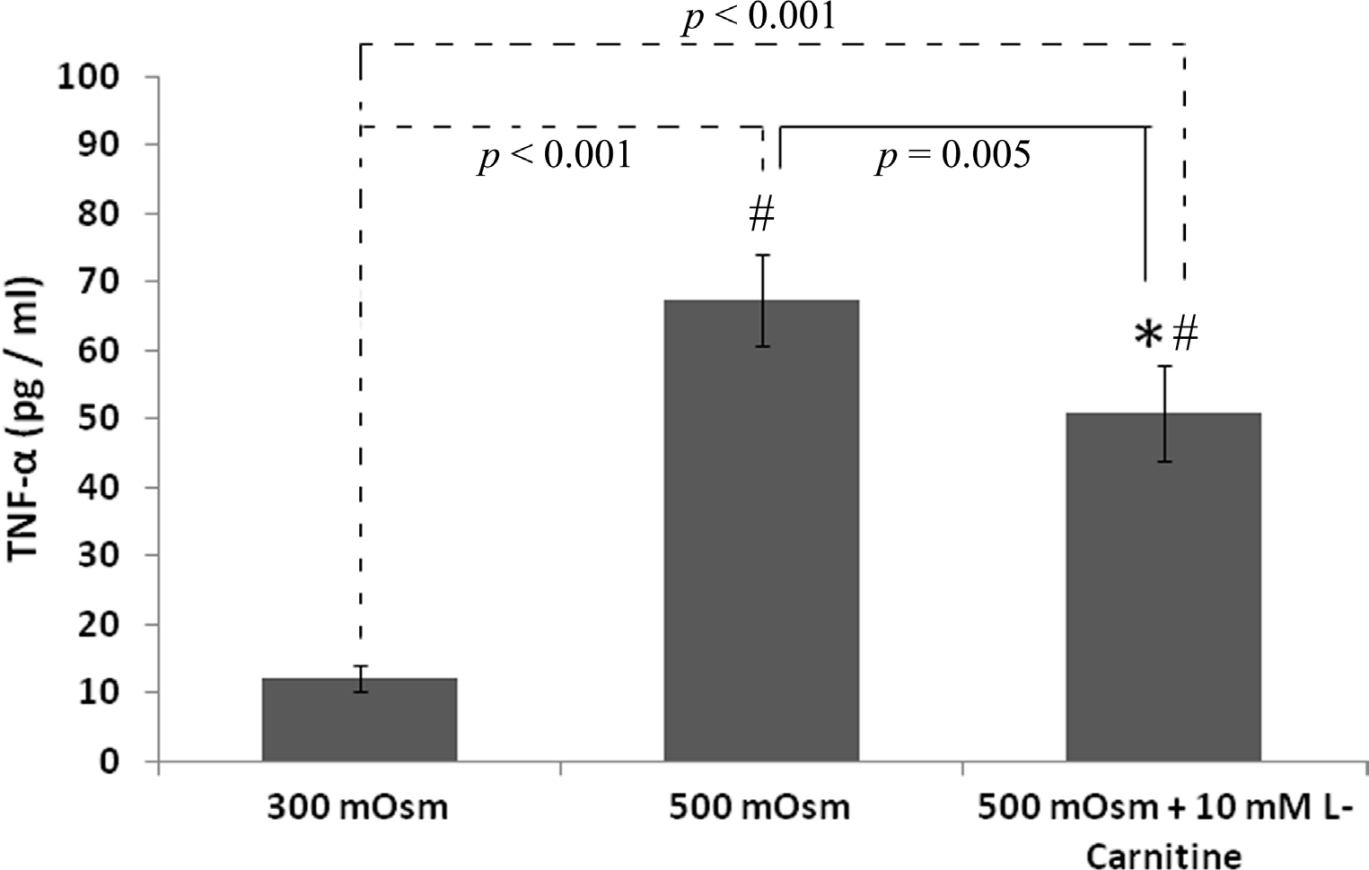
Production of tumor necrosis factor-α by human corneal-limbal epithelial cells in response to isotonic (300 mOsm) or hyperosmotic stress (500 mOsm) with or without carnitine (10 mM) for 16 h. Data represent the mean±SD of five samples. * shows the significant difference between cells treated with and without L-carnitine during exposure to 500 mOsm medium osmolarity. ^#^ shows the significant difference from 300 mOsm medium.

Exposure of HCLE cells to 500 mOsm media resulted in a significant increase in TNF-α production (six-fold, p<0.001). This increase was counteracted significantly but not fully by the addition of L-carnitine during the hyperosmotic exposure (p=0.005; Figure 2).

### Induction of cell apoptosis by hyperosmotic stress in the presence of L-carnitine

**Figure 3:**
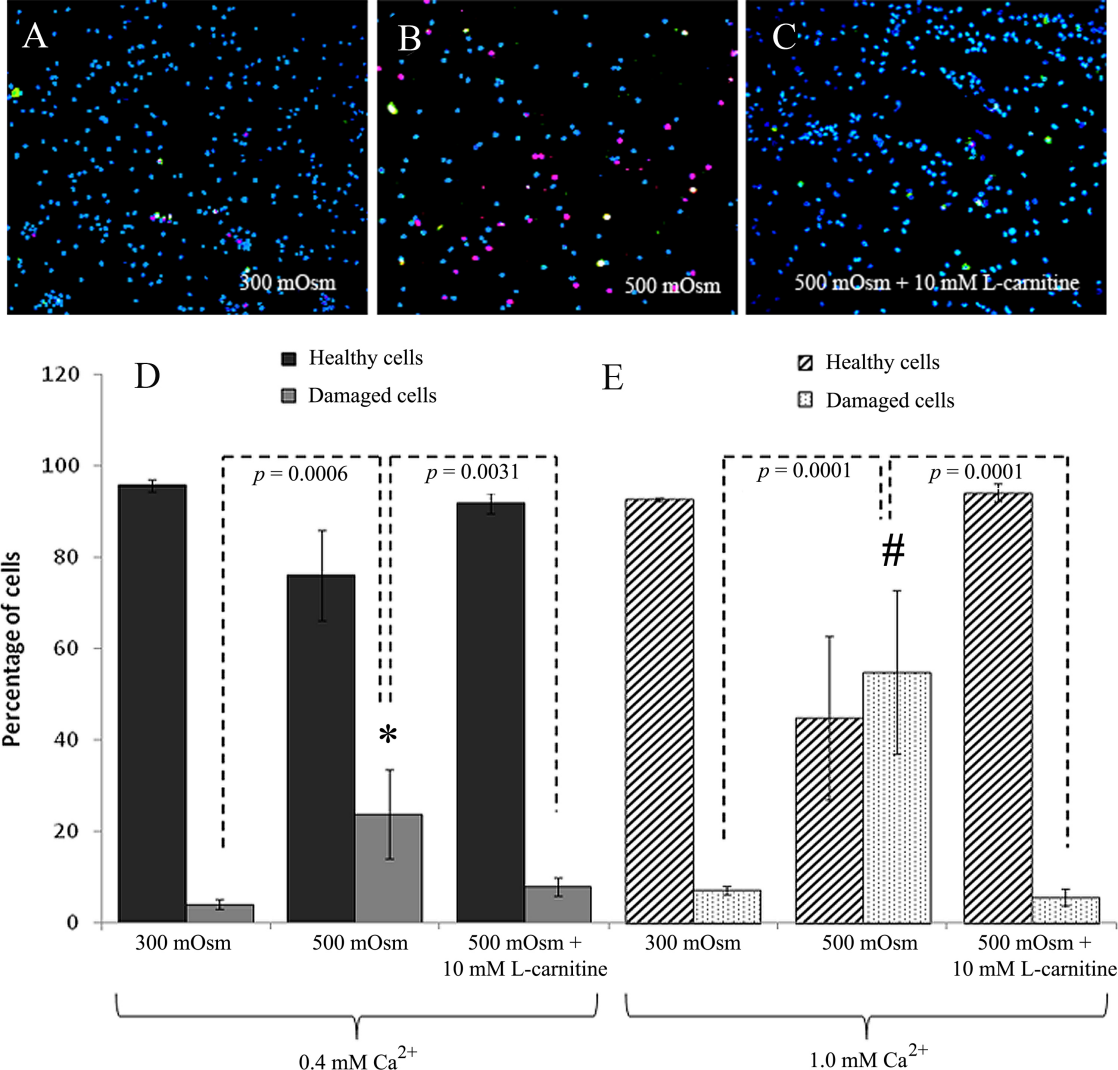
Confocal microscopic evaluation of hyperosmotic stress in the presence of 10 mM L-carnitine on the physiological state of HCLE cells. Representative images of HCLE cells stained with Hoechst (blue), annexin V (green) and PI (red) after 16 h exposure to 300 mOsm (**A**), 50 mOsm (**B**) or 500 mOsm+10 mM L-carnitine (**C**) media are shown. These images were acquired under the normal culture condition for HCLE with Ca^2+^ concentration of 0.4 mM.The percentage of healthy and damaged/dead HCLE cells under the Ca^2+^ concentration of 0.4 mM (**D**) or 1.0 mM (**E**) is shown. * and ^#^ represents a statistically significant difference between 500 mOsm and 500 mOsm+L-carnitine or isotonic (300 mOsm) conditions with Ca^2+^ concentration of 0.4 and 1.0 mM, respectively.

When the HCLE cells were exposed to isotonic (300 mOsm) media, confocal microscopic evaluation revealed a dominant population of healthy cells (Figure 3A), blue cells stained positive with Hoechst 33342 but not with annexin V or PI. Hyperosmotic stress (500 mOsm) resulted in an increase in the proportion of damaged/dead cells composed of early apoptotic (green: positive annexin V staining); late apoptotic [yellow: positive annexin V (green) + positive PI staining (red)]; and necrotic (red: positive PI staining) cells (Figure 3B). Adding L-carnitine to the hyperosmolar medium resulted in a significant decrease in the number of damaged/dead cells (i.e., those stained with annexin V and/or PI; p<0.05; Figure 3C,D).

After the cells were transferred to higher calcium-containing media, confocal microscopic evaluation revealed a heterogeneous mixed population of differentiated and undifferentiated cells (data not shown). After the cells were exposed to hyperosmotic medium (500 mOsm) in the presence of 1.0 mM Ca^2+^, the percentage of apoptotic and necrotic cells increased significantly (p=0.0001). With addition of 10 mM L-carnitine, however, a significant reduction in the percentage of apoptotic and necrotic cells was observed compared to that without treatment with L-carnitine (Figure 3E). These results were similar to that observed when the cells were cultured under low Ca^2+^concentration (0.4 mM; Figure 3D), suggesting that L-carnitine is also effective for more differentiated cells.

**Figure 4:**
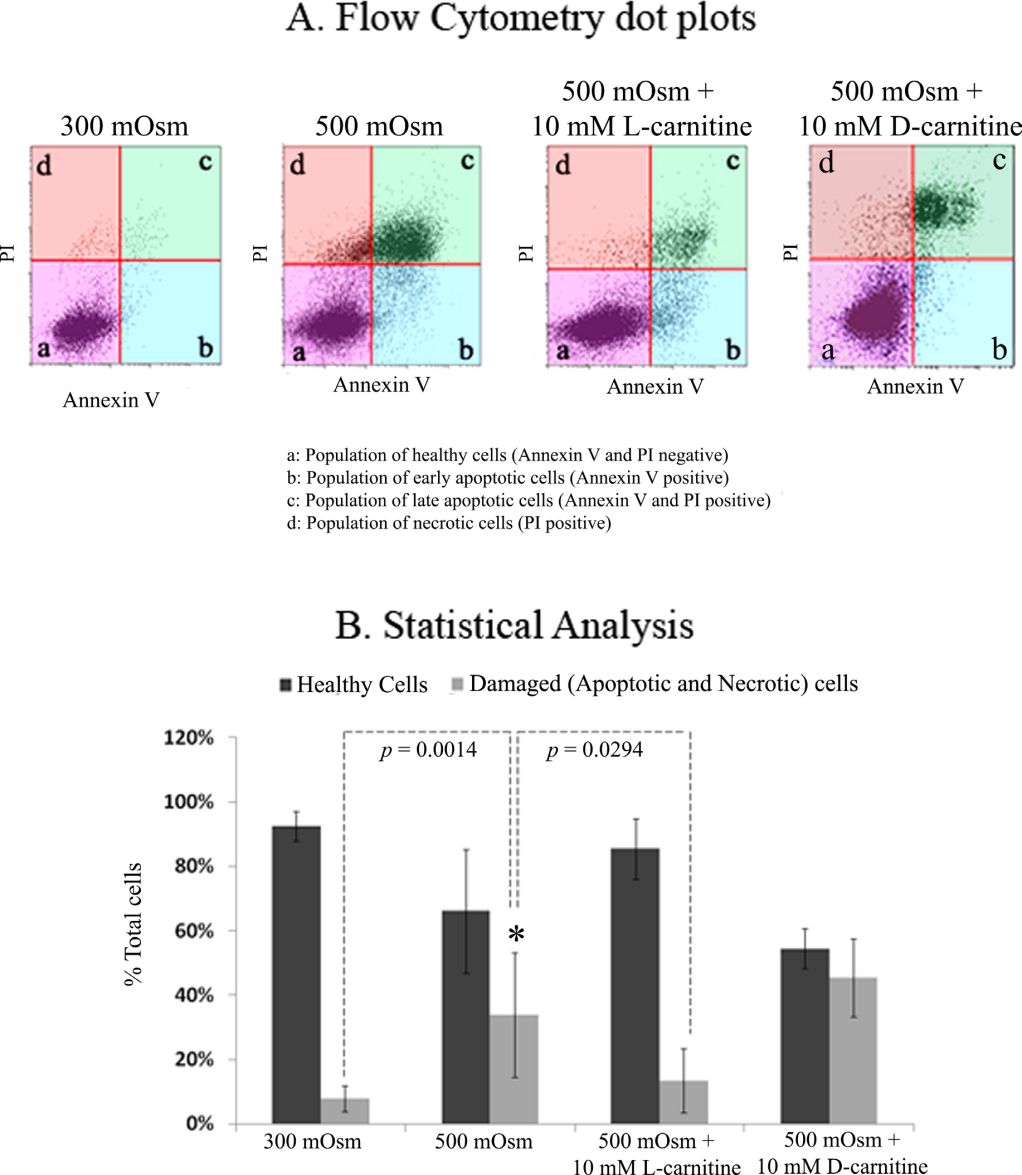
Flow cytometry analysis of the physiologic state of cells in response to isotonic (300 mOsm) media or hyperosmotic stress (500 mOsm) in the presence or absence of L-carnitine or D-carnitine (10 mM). **A**: Representative flow cytometry dot plots are shown. Quadrant ‘a'=the population of viable healthy cells that stained with neither propidium iodide (PI) nor annexin V. Quadrant ‘b’=the population of cells staining with annexin V but not PI, indicating an early apoptotic population. Quadrant ‘c’=the population of cells stained with annexin V and PI, indicating a late apoptotic population. Quadrant ‘d’=the population of cells staining positive for PI but not annexin V, indicating the complete loss of the cell membrane and thus representing necrotic cells. **B**: Statistical analysis of percentage of healthy and damaged/dead HCLE cells is shown. Data represent the mean±SD of eight samples. * represents the statistically significant difference between 500 mOsm and 500 mOsm + L-carnitine or isotonic (300 mOsm).

Further analysis using flow cytometry (Figure 4) indicated the percentage of apoptotic and necrotic cells (Figure 4A, quadrants b, c, d) was comparatively high under hyperosmotic stress (500 mOsm) compared to the isotonic control (300 mOsm), and was visibly reduced after 10 mM L-carnitine was added, but not D-carnitine, to the hyperosmolar media (500 mOsm; Figure 4A).

Further statistical analysis showed that the percentage of damaged (apoptotic and necrotic) cells when the cells were exposed to hyperosmolar medium increased significantly from 7% in 300 mOsm to 33% in 500 mOsm (p=0.0014; Figure 4B). In the presence of L-carnitine, the percentage of hyperosmolar medium (500 mOsm)–induced apoptotic and necrotic cells was reduced significantly to approach that under isotonic conditions (from 33% to 13%, p=0.0294; Figure 4B). There was no statistically significant difference in the percentage of damaged cells in response to the exposure to hyperosmotic medium with or without the presence of D-carnitine (p>0.05; Figure 4B).

### L-carnitine modulation of cell volume

**Figure 5:**
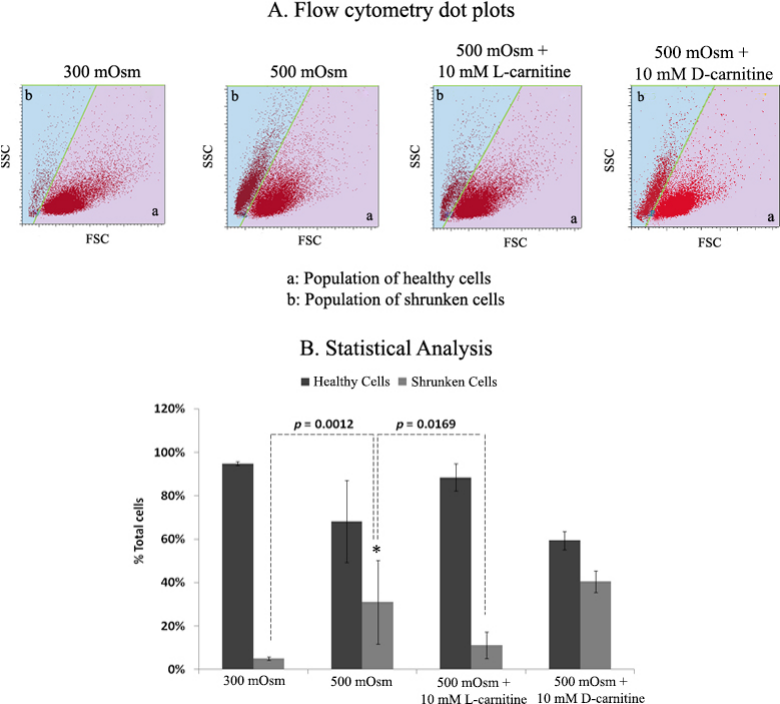
Flow cytometry evaluation of human corneal-limbal epithelial (HCLE) cell volume changes in response to isotonic (300 mOsm) medium or hyperosmotic medium (500 mOsm) with or without the presence of L-carnitine or D-carnitine (10 mM). **A**: Representative dot plots, (a) population of healthy cells and (b) population of shrunken cells, are shown. **B**: Percentage number of healthy and shrunken HCLE cells is shown. Data represent the mean±SD of eight samples. * shows the significant difference in the percentage number of shrunken cells between 500 mOsm and 500 mOsm + L-carnitine or isotonic (300 mOsm).

Flow cytometry analysis was used to determine cell volume changes in response to hyperosmolarity with or without L-carnitine. Figure 5A shows the population of cells with compromised cell volume (i.e., shrunken cells) in the presence of 500 mOsm media compared to those under isotonic conditions (300 mOsm). The percentage of shrunken cells increased from 5% to 31% (p=0.0012; Figure 5B) in the presence of the hyperosmolar media. When the cells were supplemented with L-carnitine during hyperosmotic stress, the percentage of hyperosmotic stress–induced shrunken cells decreased significantly from 31% to 11% (p=0.0169; Figure 5B). The percentage of shrunken cells after treatment with 500 mOsm + 10mM L-carnitine was not significantly different from that for the control 300 mOSm treatment (p>0.05; Figure 5B). Adding D-carnitine to hyperosmotic medium had little effect on the percentage of shrunken cells compared to that in the absence of D-carnitine (Figure 5).

## Discussion

Tear hyperosmolarity is a central factor in dry eye–related ocular surface damage, inflammation, and symptoms [11]. The key new finding of this study is that cultured human corneal epithelial cells can adapt to hyperosmotic stress by partially restoring the cell volume through uptake of a compatible osmolyte, L-carnitine, and to counteract the deleterious effects of hyperosmolarity (500 mOsm). L-carnitine regulated the volume of HCLE cells under hyperosmotic conditions, eliciting a concomitant reduction in the caspase cascade that is an inducer of apoptosis. Incubating HCLE in hyperosmolar media resulted in an increased number of shrunken, apoptotic and necrotic HCLE cells, accompanied by increased production of TNF-α and elevated activity levels of caspases-3/7, -8. and -9. Osmotic shrinkage acting as a signal to apoptosis has been observed in various cell types including human corneal epithelial cells [33-35,54,55]. A link between hyperosmotic stress and the induction of apoptosis in cultured human ocular surface epithelial cells has been established by several researchers [33-35]. Proposed pathways include the release of cytochrome c from mitochondria that then triggers activation of c-Jun N-terminal kinase, extracellular signal-regulated kinase mitogen-activated protein kinase, and caspase 3 [33] and activation of polo-like kinase 3 (Plk3) [18]. Alternative proposals include a caspase-independent pathway through the translocation of apoptosis-inducing factor from the mitochondria to the nucleus where apoptosis-inducing factor binds to DNA and leads to chromatin condensation and cell death [34].

The caspase family plays an important role in apoptosis. Two major pathways have been identified for induction of apoptosis [56,57]. One involves participation of the TNF family of receptors characterized by so-called death domains on their cytoplasmic tails. The ligation of death receptors by their ligands activates caspase-8. The other pathway involves the participation of mitochondria, which releases cytochrome c to activate caspase-9. Caspases-8 and -9 subsequently activate the downstream effector caspases, such as caspase-3/7, which are responsible for the proteolytic cleavage of a broad spectrum of cellular targets ultimately leading to cell death. In the present work, the activity of caspase-8, -9, and -3/7 of the HCLE cells subjected to 500 mOsm for 16 h increased significantly, compared to the value of isotonic cells. These data suggest that the mitochondria and death receptor pathways might be involved in the hyperosmolarity-induced apoptosis of HCLE cells.

TNF-α is a potent cytokine produced by many cell types including human corneal epithelial cells [58,59]. TNF-α is induced via activation of nuclear factor of activated T-cell-5 (NFAT-5), an important tonicity response gene regulator and survival factor in hyperosmolar stressed human limbal epithelial cells [27]. TNF-α elicits a broad spectrum of cellular responses, including cell proliferation, differentiation, and apoptosis [60,61]. TNF-α triggers apoptosis by binding to the TNF-α receptor and subsequently activates caspase-8 [62], which forms a death-signaling complex with tumor necrosis factor receptor type 1-associated death domain protein (TRADD), and Fas-associated protein with death domain (FADD) that can further induce effector caspases such as caspase-3/7 causing initiation of apoptosis [63,64]. In agreement with others [55,58], we also observed the induction of TNF-α expression by hyperosmotic stress, and we found the increased TNF-α expression was associated with the increased population of apoptotic/necrotic cells subjected to prolonged exposure to hypersomotic stress.

Many types of cells respond to extracellular hyperosmolarity by the accumulation of organic osmolytes, such as taurine and betaine, which protect cells from the perturbing effects of high extracellular concentrations of electrolytes [65-68]. A role for L-carnitine as a stress protectant has been described in which L-carnitine protects against stress activation of MAP kinases in cultured corneal epithelial cells [17]. Previous studies have shown that HCLE cells have the specific transporters OCTN1 and OCTN2 for L-carnitine [49] and the uptake of L-carnitine is Na^+^-dependent [69], which suggests that the uptake of L-carnitine by HCLE cells might be facilitated in a hyperosmolar environment similar to that of other osmoprotectants such as betaine and taurine whose uptake can be facilitated and increased under hyperosmolar conditions [66,69]. Although L-carnitine uptake by corneal epithelial cells appeared to be maximal at 0.5 mM under iso-osmolar conditions [69], 10 mM L-carnitine was used in the present study to ensure an excess of L-carnitine in the hyperosmolar culture medium (500 mM). Other researchers have also shown that L-carnitine at 10 mM is effective as an osmoprotectant for human primary corneal epithelial cells [17]. In the present study, we demonstrated that HCLE cells in the presence of L-carnitine reduced the hyperosmolarity-induced cell shrinkage and subsequent cell apoptosis, therefore enabling increased survival under conditions of high (external) osmolarity.

L-carnitine is a zwitterionic molecule. Unlike many other zwitterionic amino acids that are active participants in cellular metabolism and thus the intracellular concentrations required for their effective osmoprotection are impossible to achieve, L-carnitine is not typically metabolized [39] and has been demonstrated to act as an osmoprotectant in various mammalian systems and bacteria [39,70-72]. We characterized the properties of L-carnitine as an osmoprotectant particularly in restoring cell volume and protection against hyperosmolarity-induced cell apoptosis. L-carnitine’s ability to regulate hyperosmolarity-induced cell volume changes might be one of the underlying mechanisms by which L-carnitine suppresses the initiation of hyperosmotic stress-induced apoptosis, and contributes to the survival of cells, although not fully, under hyperosmolar conditions. D-carnitine (known to be biologically inactive [73]), however, did not exhibit similar osmoprotection activity. D-carnitine’s inability to stabilize cell volume under hyperosmotic stress corresponded with the observation of a lack of protection for cells under hyperosmotic stress, further supporting the mechanism of action for L-carnitine as an osmoprotectant: regulate cell volume under hyperosmotic stress and ameliorate hyperosmotic stress–induced cell apoptosis.

L-carnitine is antiapoptotic [74,75] and antioxidative [76], protects Jurkat cells against Fas-mediated apoptosis, and is a potent inhibitor of caspase-3 [77]. L-carnitine inhibits the oxidative stress-induced activation of caspase-3 in a dose-dependent manner [76]. Carnitine also reduces the quantity of oxidant-induced cytosol cytochrome c and increases antiapoptotic Bcl-xL expression, resulting in protection of cardiomyocytes from doxorubicin-induced apoptosis [74]. We found that adding L-carnitine significantly reduced the activity of caspase-9 presumably not directly but as the result of preventing activation in HCLE cells during hyperosmotic stress. Further, L-carnitine also inhibits Fas-induced apoptosis [78], which can lead to inhibition of caspase-8 [77]. Interestingly, we found L-carnitine inhibited hyperosmolarity-induced TNF-α expression but showed little effect on the activity of caspase-8 under the study conditions. Caspase-8 is best characterized as the principal upstream caspase of death receptor signaling, and evidence indicates caspase-8 is implicated in non-receptor-mediated apoptosis [79]. In some cell types, lack of caspase-8 does not protect completely from TNF-induced cell death. TNF-α signaling uses caspase effectors in addition to caspase-8 [79].

Therefore, we speculate that the extrinsic caspase-8 dependent pathway might play a minor role for HCLE cells at times of hyperosmotic stress, at least in vitro under our study conditions where 10 mM L-carnitine was tested. Whether this concentration is optimal for studying the interactions of L-carnitine with caspase pathways has yet to be determined, and further investigation is needed to determine the precise mechanism of cell death that results from hyperosmolar stress on corneal epithelial cells and the role of L-carnitine protection from cell death. In conclusion, the present study confirmed that L-carnitine can regulate human corneal epithelial cell volume under hyperosmotic stress and ameliorate the initiation of hyperosmotic stress-induced apoptosis.
